# Phytochemical composition of *Clinacanthus nutans* based on factors of environment, genetics and postharvest processes: A review

**DOI:** 10.37796/2211-8039.1451

**Published:** 2024-06-01

**Authors:** Nurul H. Sabindo, Rusidah M. Yatim, P. Kannan Thirumulu

**Affiliations:** a School of Dental Sciences, Universiti Sains Malaysia, 16150 Kubang Kerian, Kelantan, Malaysia; b Division of Research and Innovation, Level 2, Chancellory II, Building E42, Universiti Sains Malaysia, USM, 11800 Pulau Pinang, Malaysia; c Human Genome Centre, School of Medical Sciences, Universiti Sains Malaysia, 16150 Kubang Kerian, Kelantan, Malaysia

**Keywords:** Chemical constituent, *Clinacanthus nutans*, Geographical distribution, Phytochemicals, Phytochemistry

## Abstract

*Herpes simplex*, varicella-zoster lesions, skin rashes, diabetes, snake bites and insect bites have all been treated by using *Clinacanthus nutans* (*C. nutans*). The pharmacological effects of *C. nutans* are influenced by the presence of terpenoids, flavonoids, alkaloids, phenolic acids, saponins, glycosides, steroids and tannins. This review focused on the phytochemical makeup, which varies geographically and is a subject of scarcely existing knowledge. *C. nutans* served as the primary search term, while the keywords “phytochemicals”, “chemical component” and “phytochemistry” were used to search the literature in the Google Scholar, PubMed, Scopus and Web of Science databases. The articles pertinent to the subject were found and reviewed. The phytochemical composition of *C. nutans* varied depending on the region it was cultivated in, and was influenced by the environmental conditions, genetics, air temperature and postharvest practices.

## 1. Introduction

Because of curved shape of the plant's stem, which resembles the curvature of an elephant's trunk, *Clinacanthus nutans* is known as “Belalai Gajah” in Malaysia [1]. Malaysia, Indonesia, Thailand, Vietnam and China are all home to *C. nutans*. It is used to treat liver cancer, diabetes, lesions caused by varicella-zoster, insect bites, snake bites, skin rashes and *Herpes simplex* [2]. Alkaloids, steroids, terpenoids, flavonoids, saponins, glycosides, tannins and phenolic acids are some phytochemicals found in *C. nutans* that contribute to their pharmacological effects. However, depending on their environment, plants of the same species may have different phytochemical compositions. For instance, the flavonoid and phenolic levels of *C. nutans* from Thailand were higher than those from Malaysia [3]. Uncertainty surrounds this variation in the phytochemical composition of *C. nutans*, which may be impacted by the genetic, geographic altitude, local ecological conditions and places with various environmental characteristics [1]. This review focuses on the phytochemical variations existing in *C. nutans* and to ascertain the causes underlying these variations due to the distribution of *C. nutans*. This review is aimed at having a twofold effect on the readers: first, it would serve as a resource to identify the variations in different geographical areas and to design research accordingly. Secondly, it would eliminate the need for duplicating investigations already available, thereby enabling designing studies with novel insights in this area.

*C. nutans* (Burm. f.) Lindau can be found in Southeast Asian nations which is a traditional herb and is a member of the Acanthaceae family. The taxonomy of *C. nutans* can be categorized as follows: *C. nutans* is a member of the Phylum - Tracheophyta, Class -Magnoliopsida, Order - Lamiales, Family - Acanthaceae, Genus - *Clinacanthus*, and Species - *nutans* [4]. The stems of *C. nutans* are cylindrical, striate, hairless and they can grow as tall as one metre. The stems are short, fragile, slender, and slightly curved, giving them the appearance of an elephant trunk [5,6]. The leaves are oblong or lanceolate shaped on the side opposite the narrow elliptic leaf blades. When a leaf is young, its surface is pubescent; as it becomes older, however, it turns glabrescent. At the top of branchlets and branches, yellowish blooms proliferate in clusters [7].

## 2. Phytochemical composition of *C. nutans*

In recent decades, plant research has expanded significantly. Both secondary and primary metabolites are present in plants [8]. Primary metabolites are those chemical elements that are naturally present in organisms and directly contribute to plant growth. The bioactive phytochemicals, on the other hand, are produced by plants in response to conditions of the environment called secondary metabolites and are believed to provide extra health advantages [3]. Since plants are stationary autotrophs, they must evolve coping mechanisms for a number of problems, including self pollination, seed distribution, solar radiation, nutritional shortages, pathogens' cohabitation and herbivores in the surroundings [9]. Phytochemical screening is limited to functional groups derived from plants. In-depth investigations are required to identify particular bioactive substances. Liquid chromatography mass spectrometry (LCMS), thin layer chromatography (TLC), gas chromatography mass spectrometry (GCMS) and high-performance liquid chromatography (HPLC) are the techniques that are most frequently used to separate and identify the phytochemical compounds [3]. These methods can help to direct the class as well as the functional groups that make up a plant's chemical composition.

Based on the origin, the different chemical classes present in *C. nutans* extracts according to a specific phytochemical screening test are summarized in Table 1. Flavonoids, steroids, phytosterols, triterpenoids and glycosides are typically the most commonly found phytochemical classes in *C. nutans* extract when the plant is subjected to extraction employing polar solvents like water, ethanol, aqueous organic solvent, methanol or semi polar solvent. The different metabolites identified in *C. nutans* are presented in Table 2. The quantity of all the aforementioned phytochemicals in *C. nutans* is also influenced by the plant's provenance and post harvesting methods. Under different climatic conditions, plants of the same species may have slightly different secondary metabolite concentrations [1,3,6,8].

### 2.1. Phenolic acids and flavonoids content

Phenolics, which include hydroxyl groups connected to aromatic rings, are one of the main categories of plant's secondary metabolites [3]. The two subclasses of phenolics are flavonoids and non-flavonoids. Simple phenols, coumarins, benzophenones, stilbenes, acetophenones, hydrolysable tannins, phenylacetic acids, lignans, cinnamic acids and benzoic acids are examples of non-flavonoids [3,10]. Flavonoids include anthocyanins, proanthocyanidins, flavones, flavanones, dihydroflavonols, flavan-3-ols, flavonols, isoflavones and chalcones [3,10]. Due to the phenolics' health benefits, which include antioxidant, anti-inflammatory [3,11], estrogenic, enzyme inhibition, antimicrobial, antiallergic and anticancer activities [12] interest in ingestible naturally obtained phenolic rich plant sources has increased recently. The four phenolic substances that Wong et al. [13] sought to examine in *C. nutans* were gallic acid, caffeine, catechin and quercetin; unfortunately, they only found quercetin. The content of isovitexin, orientin, schaftoside and vitexin in *C. nutans* leaves collected from Sendayan (Negeri Sembilan), Kota Tinggi (Johor) and Taiping (Perak) was investigated [14]. They found that *C. nutans* from Taiping, Perak, Malaysia had the greatest concentration of all the four phenolics.

The Aluminium chloride (AlCl_3_) method was used to determine the flavonoid content present in *C. nutans*. The reaction of AlCl_3_ with carbonyl as well as the hydroxyl groups in flavonoids in alkaline solution is the primary method for identifying flavonoids [8]. Chelyn et al. [14] found the difference in total flavonoid content (TFC) observed with the variety in flavonoids, notably flavone C-glycosides, such as schaftoside, isovitexin, orientin and vitexin found in leaves of *C. nutans* obtained from different sources in Malaysia was similar with the variation in TFC as reported by Fong [3].

### 2.2. Other contents

When *C. nutans* is extracted with water, ethanol, methanol or any aqueous organic solvent, additional phytochemical groups such as steroids, triterpenoids, glycosides and phytosterols are frequently discovered. The existence of saponins, tannins and alkaloids is influenced by the plant's origin and post harvesting technique [8]. Alkaloids were found in water or chloroform leaf extracts, in contrast to aqueous methanol or methanol extracts of *C. nutans* leaves that were obtained in Malaysia [8]. Malaysian methanol extracts, however, did contain saponins, which was absent in water extract from Indonesia, as well as chloroform and aqueous methanol extracts from Malaysia [8]. Tannins were found in water, aqueous methanol and chloroform leaf extracts but was absent in methanol extract [8].

Mustapa et al. [15] analyzed the total phytosterol (TP) and β-sitosterol by UV–vis spectrophotometry and HPLC, respectively and reported that microwave assisted extraction (MAE) pretreatment resulted in higher content of TP and β-sitosterol than the Soxhlet extraction method. They also reported that a remarkable content of TP and β-sitosterol was obtained by supercritical carbon dioxide extraction which was higher than the concentrations obtained by MAE or Soxhlet extraction method.

Compared to stems that are mature or samples that had been maintained for a duration of four days, young leaves or plant samples had elevated chlorophyll concentrations [16]. Harvests of immature *C. nutans* leaves also contained more ascorbic acid. When stored for 4 day as opposed to one day, the ascorbic content of the recovered *C. nutans* leaves was found to decrease [8]. However, it was discovered that the absolute ethanol aerial extract of *C. nutans* contained more chlorophyll than the absolute acetone aerial extract [15].

## 3. Factors that influence variations in phytochemical constituents in *C. nutans*

Even among plants of the same species, secondary metabolite concentrations can differ. This is because the environmental conditions which include humidity, rainfall, water fluctuation, temperature, exposure to microbes in the soil, changes in pH value of soil and nutrients have a significant impact on the quantity and quality of medicinal plants' secondary metabolites [1]. The environment in which plants thrive also plays a key role in the production of phenolic compounds. This can contribute to the variations in these compounds’ medicinal efficacy [3]. Certain substances can only be synthesized or significantly enhanced in specific environments [3,31]. It is established that medicinal plants of the same species grow differently depending on altitude, air temperature and sunlight [3].

### 3.1. Environmental factors influence

Soil affects plant growth and development, and secondary metabolites accumulation is highly reliant on soil water stress (drought stress), soil salinity and soil fertility [32]. Many studies have shown that plants subjected to drought stress accumulate a higher concentration of secondary metabolites than those grown in wellwatered circumstances [33]. In Ismail et al. [1] study, they selected eight samples of *C. nutans* from different locations in Malaysia which were Pongsu Seribu (PPS), Tasek Gelugor (PTG), Batu Maung (PBM), Batu Ferringhi (PBF), Jeniang (KJN), Sungai Petani (KSP), Kuala Ketil (KKK) and Sungai Batu Pahat (SBP). Locations PBF and KKK of *C. nutans* demonstrated high levels of total phenolic content with acidic and neutral pH levels in the soil, respectively. Both locations had an excessive amount of phosphorus in their soil. The antioxidant activity may be related to the soil low nitrogen content and the location sites moderate soil moisture levels. The highest antioxidant activity was found in *C. nutans* at the study sites PPS, PTG, and KKK, which also had the lowest nitrogen levels and middle levels of soil moisture of all the locations. Their findings indicated that *C. nutans* could be successfully grown in soil with an intermediate (normal) moisture level, one that was neither too dry nor too wet [1].

Studies have suggested that UV-B irradiation from sunlight has a role in the activation of the biosynthesis of phenylpropanoid and flavonoid compounds [34,35]. The formation of reactive oxygen species (ROS) by UV-B radiation causes damage to DNA and other macromolecules in plant cells [36]. Thus, phenolic and flavonoid molecules in plants generated by UV-B exposure serve as UV-B-protective chemicals, as they have a high UV spectrum absorption and potent antioxidant activity [37]. Kong et al. [38] analyzed the phytochemical content on *C. nutans* leaves and stems from different areas which were Yik Poh Ling (YPL) unshaded sample (exposed to direct sunlight), and You Dun Chao (YDC) unshaded and shaded (with black shade netting) samples. They found that all the samples contained alkaloids, flavonoids, glycosides, saponins, steroids, terpenoids, phenols and tannins. However, the unshaded leaves of *C. nutans* from YDC exhibited significantly higher antioxidant properties (966.00 mg GAE/g of dried sample in total phenolic content) than the other samples (P < 0.05) [38].

Plants under oxidative stress from low air temperatures produce more ROS. Plant's ROS harm cells via interaction with DNA, proteins, and lipids [3,39]. By producing antioxidants such as phenolic compounds, which include flavonoids, plants use protective mechanisms to offset the harmful effect [3,40]. Low temperatures affect plant biochemical processes by altering the gene expression engaged in phenolic compound synthesis, for instance, the phenylalanine ammonia-lyase (PAL) and chalcone synthase (CHS) genes [3]. Low temperatures increase the activity of the PAL and CHS enzymes in plants, leading to an increase in phenolic compounds and their glycoside and ester-bound forms [3,41]. Sanders also pointed out that areas with higher levels of unsaturated fatty acids are typically colder and that these acids produce antioxidants that support the body's defense against environmental stress [42]. Phenolics play a key role in plants' ability to scavenge hydrogen peroxide, including glutathione, ascorbic acid and peroxidase. More of these compounds must be created in order to safeguard plants grown there because this mechanism is less effective at low temperatures [43]. According to Fong [3], samples from Thailand varied more in the total phenolic content (TPC) than those from Malaysia. Different elevations of *C. nutans* samples taken from Sandakan, Malaysia showed noticeably greater TPC levels. Rather than growing at lower elevations in warmer air, these were cultivated at higher altitudes in cooler air. Similar to the TPC, Fong found that samples from regions with higher elevations and cooler air temperatures contained more flavonoids [3]. In general, higher elevation plant growth is correlated with higher concentrations of phenolic compounds due to increased ultraviolet radiation intensity and cooler air temperatures [3]. However, higher elevations do not generally have more phenolic compounds [44]. The discrepancy in the results suggests that altitude fluctuation affects phenolic content differently for each species and plant, or that it could be caused by soil nutrients [3]. Elevation alterations could be viewed in terms of adaptive responses or a plant's mechanism of defence in response to the increased levels of UV-B radiation that are present at greater elevations [45]. Numerous plant species have shown that increasing UV-B light exposure makes contact hypersensitivity, the main enzyme participating in the initial step of synthesis of flavonoids, more active [46]. Light-absorbing flavanols, flavonoids, and anthocyanins, especially act as a sunscreen to protect the internal tissues of leaves and stems from ultraviolet B radiation damage by accumulating in epidermal cells [47].

Fong [3] compared TPC and TFC of *C. nutans* samples from 11 different locations in Thailand, Malaysia, and Vietnam, each with its own specific climate and geographic characteristics by using Folin-Ciocalteu method and HPLC methods. *C. nutans* samples were collected from Sabah (elevation ranging from 6.8 m to 158.7 m; mean annual temperature from 22.9 °C to 30.8 °C and mean annual rainfall from 1975.0 mm to 2973.0 mm) and Negeri Sembilan (elevation ranging from 66.3 m to 83.4 m; mean annual temperature from 22.4 °C to 31.3 °C and mean annual rainfall from 2010.0 mm to 2124.0 mm) in Malaysia. In Thailand, the samples were collected from Nakhon Pathom (elevation ranging from 4.3 m to 8.1 m; mean annual temperature from 22.7 °C to 32.7 °C and mean annual rainfall from 1237.0 mm to 1334.0 mm) and Chiang Mai (elevation ranging from 309.5 m to 439.4 m; mean annual temperature from 18.7 °C to 31.5 °C and mean annual rainfall from 1191.0 mm to 1261.0 mm). In Vietnam, the samples were collected from Ho Chi Minh (elevation at 2.2 m; mean annual temperature ranging from 23.1 °C to 31.9 °C and mean annual rainfall at 1873.0 mm). It was concluded that *C. nutans* samples grown at higher elevations with cooler air temperatures showed higher TPC than those grown at lower elevations with warmer air temperatures [3].

### 3.2. Influence of genetics

The quality and quantity of phenolic compounds are affected by genetic factors [3]. For instance, the expression of the genes F3H and ANR, which are involved in the synthesis of flavonoids, regulates the pattern and level of production of these compounds [3]. Genetics rather than environmental elements like climate and sunlight play a significant role in determining the phenolic content of some plant species [48,49]. A synergistic interplay of genetic and environmental factors may have an impact on plant phenolic production and accumulation [3]. Samples of *C. nutans* obtained in 11 different Malaysian, Thai, and Vietnamese locations were studied genetically [3] and the genetic similarity of most samples made them likely to be clones, and there was minimal variability between the different countries.

Ismail et al. [50] analyzed the genetic diversity of eight populations of *C. nutans* in the northern area of Peninsular Malaysia. The genetic diversity revealed a significant percentage of variation at the species level as compared to the population level. Moreover, molecular variance analysis demonstrated that genetic diversity within populations of *C. nutans* was larger than genetic diversity among populations [50]. In the study, it was found that the SBP population (situated in Taman Herba Perlis, a commercial herbal garden in Peninsular Malaysia's northern west coast) displayed the largest genetic variation in comparison to other populations, according to Nei's genetic diversity index. This finding agreed with Gao et al. [51] findings that populations in long-maintained, well-established gardens had the most genetic diversity. This is because many gardens have over the years preserved several collections from various wild populations, made possible by the presence of a community and a human society in that area [51]. PBM Population exhibited limited genetic diversity because it was located in a cultivated farm, whereby extensive harvesting operations prevented *C. nutans* from reaching maturity, hence inhibiting the formation of flowers, which are needed for cross-pollination [50]. This contrasts with other Acanthaceae species, particularly herbaceous species that may be easily disseminated from seeds, such as *Ruellia nudiflora*, which had higher genetic variation but was cultivated in disturbed open spaces and diverse agricultural areas [52].

The high genetic diversity at the species level is caused by many things, such as a recent drop in population size that did not give time for isolation to spread [53]. Geographical distribution is one of the determinants of a species' genetic diversity. The Acanthaceae family consists primarily of widely dispersed herbaceous plants and shrubs. *C. nutans* has been found to be widely dispersed in South China, Thailand, Vietnam, Malaysia and Indonesia [14]. It can be found in both natural and cultivated habitats, such as grasslands, hillsides, shrubs, valleys, coastal regions, dense and open forests. *C. nutans* has a wide geographic distribution and is distinguished by its high genetic diversity at the species level but only moderate genetic diversity at the population level. As commercial plantings of *C. nutans* practice vegetative propagation through stem-cutting, this genetic diversity will further deteriorate. In the long-term, the modest diversity at the population level, frequent harvesting before maturity, and vegetative propagation may further lower the genetic diversity at the population level and species level as a result of the loss of rare alleles [54]. Based on the similarity matrix, populations PTG, KSP, KKK, and PPS of *C. nutans* displayed significant genetic similarity and low genetic distance. These populations are dispersed throughout lowland areas and are subjected to rigorous farming methods. Conversely, populations PBF and PBM exhibited a low genetic similarity and considerable genetic distance, which is most likely a result of the disparate growth environment and farming practices. The PBF population is concentrated on the northern coast's hilly coastal region, whereas the PBM population is concentrated in the southern coast's agricultural plains [50]. Hence, Fong [3] concluded that there was a lack of genetic diversity in the species of *C. nutans* despite them being geographically distant, and thus, it should be multiplied by sexual reproduction which will result in increasing its genetic diversity and in turn help in the long-term survival of the species.

### 3.3. Postharvest processes influence

Postharvest describes the process by which a product moves from the stage of harvest to the level of readiness for usage. Maximizing drying, storage and extraction to retain bioactive components are the main objectives of medicinal plant postharvest activities [3]. Among these are the drying and extraction processes. Drying medicinal plants is vital to extending their storage shelf life since it lowers the moisture content and prevents degradation and infestation [3]. Convective drying is widely used to dry plants since it has quicker drying times and more precise temperature control [3,55]. Fong [3] found that the TPC and TFC concentrations in *C. nutans* leaves which were dried at various temperatures (40 °C, 50 °C, 60 °C, 80 °C, and 100 °C), the total contents of both phenolics and flavonoids grew as the temperature increased, however the TFC somewhat dropped at the highest temperature (100 °C). Despite, the findings of the majority of investigations on the impact of heat treatment on the phenolic and flavonoid contents are contradictory. Some studies reported an increase in the phenolic and flavonoid contents with increasing drying temperatures, including capsicum [56], goldenberry [57], stevia leaves [58] and citrus peels [59]. On the other hand, other studies found a decrease as seen in sea buckthorn [60], motherwort and peppermint leaves [55], while some found no significant changes as seen in murta berries [61] and rosemary leaves [55].

Drying temperatures have an effect on the quantity and quality of phytochemical components in plant materials [3,55,59]. According to Mrad et al. [62], a decrease in polyphenols during drying may be due to the interaction of these chemicals with other molecules, such as proteins, or changes in the chemical structure of polyphenols that cannot be extracted or identified using existing methods. Alonzo-Macias et al. [63] suggested that the reduction of TFC and the amount of phytochemical compounds in general at the highest drying temperature, could be caused by the thermal breakdown of heat-sensitive compounds. The synthesis of phenolic compounds at high temperatures, such as 80 °C and 100 °C, may be attributed to an increase in content due to the availability of phenolic precursors, resulting in non-enzymatic interconversion between the molecules [64]. Moreover, a decrease in the activity of the phenolic oxidation enzymes peroxidase (PO) and polyphenoloxidase (PPO), which are involved in phenolic oxidation, may result in an increase in the level of phenolics [65]. According to Mizobutsi et al. [66], high temperatures (between 60 °C and 100 °C) rendered both PO and PPO enzymes inactive. In order to preserve bioactive chemicals in plant materials, the right drying temperature must be chosen. By preserving their structural integrity during drying, this ensures that the chosen compounds intended chemical and therapeutic capabilities are realized [3,67].

Extraction is crucial to the phytochemical analysis and biological activity of medicinal plants since it is required to separate the desired components from the plant materials. The production of phytochemicals involves the utilization of solid–liquid, supercritical fluid, pressurized liquid and pressurized hot water extractions. Solid-liquid extraction is the most widely used method for separating phytochemical components from plant sources [68]. In this process, solvent is applied to particles, chemicals from plant matrix are desorbed, solutes are solubilized in the solvent and solute molecules are diffused into the solvent [69]. The extraction solvent system is chosen mostly based on the polarity and chemical nature of the solutes or phytochemicals being extracted. This is based on the principle “like dissolves like”. Hexane, chloroform, dichloromethane, diethyl ether, or ethyl acetate are used to extract less polar phenolic compounds (flavanols, chlorinated phenols, flavanones, isoflavones and methylated flavones). Contrarily, methanol and ethanol are used to extract more polar phenolic chemicals (vanillic acids, ferulic acids, aglycones and flavonoid glycosides) [3]. Some phenolic substances, including benzoic and cinnamic acids, permeate between solvent phases that are mutually saturated and cannot be sufficiently extracted by using only water or pure organic solvents [3]. Therefore, alcohol–aqueous combinations are suggested [70]. While organic solvents like methanol and ethanol operate as hydrogen bond acceptors and donors respectively, the solvation process is sped up by the presence of water [68]. This synergistic effect raises the chemical solubility, increasing the capacity for extraction. For example, *C. nutans* leaves and stems extracted with 50% ethanol had higher total phenolic (1.6 times) and flavonoid (2 times) contents than those extracted with 86% ethanol [3,68], demonstrating that the former concentration of ethanol is the ideal solvent combination to extract the plant's polyphenols [3]. Various plant species and even their components contain a variety of phenolics that are selectively soluble in various solvents and belong to various classes or groups. Moreover, there is a chance that phenolics will interact with other plant substances, such as proteins and carbohydrates, creating complexes with various solubilities [71]. Thus, the polarity of the solvent plays a crucial role in the extraction of phenolic compounds, and no single standard extraction method is utilized to extract all plant phenolics. Water, methanol, ethanol, acetone, and combinations of these solvents with water, with or without acid, are the most often used solvents for extracting phenolic chemicals [71–74].

Supercritical fluid extraction (SFE) is based on the solubilizing power of fluids that are kept above their critical point [75]. Supercritical fluids combine gas-like mass transfer abilities with liquid-like solvating power. Their very low surface tensions make it easy for them to get into microporous materials like herb matrices. In SFE, density is correlated to solvating power [75]. In the industry of phytochemical extraction, carbon dioxide (CO_2_) is often the SFE solvent of choice because it is non-toxic, does not react with chemicals, and is easy to get back by venting the gaseous CO_2_. However, the two disadvantages are that the required equipment is costly, and the method is not suitable for the extraction of all phytochemicals [75].

Pressurized liquid extraction (PLE), which is also called accelerated solvent extraction, is a new method that uses organic solvents at pressures of about 14 MPa and temperatures above the boiling point of the solvent [75]. PLE offers the advantage of fast extraction durations, low solvent consumption, and high extraction yields by pressurizing and working at or above boiling point solvent temperatures [76]. The increased performance with PLE is due to the following factors: the solubility of solutes increases with increasing solvent temperature; higher solvent temperatures result in higher diffusion rates; and greater disruption of the strong solute–matrix interactions [76].

Pressurized hot water extraction (PHWE) is an extraction method that uses hot/liquid water to extract the desired phytochemicals. With pressurized systems, liquid water temperatures between 1008 °C and 3748 °C, the critical temperature of water can be reached [77]. Polar phytochemicals can be extracted from plant matrices by using PHWE. The high-water temperature of PHWE is a disadvantage since it exposes the phytochemical to degradation. Nonetheless, PHWE is an inexpensive and environmentally beneficial method for botanical preparation if the targeted phytochemical can withstand a high-water temperature [75].

It is unknown if this is also true for *C. nutans*, however the solvent temperature has an impact on the quantity of phenolic compounds recovered from plants [3]. Higher solvent temperatures can lead to better analyte solubility by increasing mass transfer rate in the plant's constituents and reducing interactions in physical chemistry [3]. Higher temperatures reduce the solvents' viscosity and surface tension, allowing the plant matrix to be penetrated by the solvents [78]. For example, extracting *Andrographis paniculata* leaves with ethanol at 65 °C produced 14% more total phenolic content than at 25 °C [79]. The leaves of *Camellia sinensis*, which showed a similar pattern, had 25% more phenolic compounds when extracted with water at 90 °C than at 25 °C [80]. However, a temperature that is too high can increase the likelihood that a chemical will degrade by hydrolysis, internal redox, or polymerization, which will reduce the amount of phenolic compounds that are produced [81]. For example, anthocyanins are best extracted at temperatures between 20 °C and 50 °C because they break down quickly at temperatures above 70 °C [78].

Fong [3] found that with more polar (water) solvents, a greater overall extraction of phenolics and flavonoids from the leaves was accomplished and that the TPC and TFC concentrations were higher in the crude formaldehyde extract. This finding is consistent with earlier research on the leaves of *Mentha* spp. (Lamiaceae) [82], *Psidium guajava* (Myrtaceae) [83], *Leea indica* (Vitaceae) [84], and *Ficus carica* (Moraceae) [85], which suggested that the types of solvent used, its polarity, and the solubility of phytochemical compounds in the extraction solvents have a significant impact on the extraction efficiency and recovery of the compounds.

## 4. Conclusions

This review offers a compendium of research information supporting the influence of multiple geographical factors on the phytochemical composition of *C. nutans*. The production of phytochemicals in *C. nutans* is seen influenced by various pre- and postharvest factors pertaining to agricultural techniques, environment (microclimate, location, growing season, soil type and nutrients), plant growth and maturity, postharvest storage and processing. Furthermore, the choice of the part of the plant, its age and origin as well as methods relating to plant preparation and experimental design are also seen to influence the composition of the phytochemicals derived from *C. nutans*. Thus, this review emphasizes the need for optimizing the sampling methods and experimental techniques for the concerned geographical location for ensuring repeatability in the consistency in the phytochemical compounds from *C. nutans*. In as much as geographical factors are seen to influence the phytochemical composition of *C. nutans* in the areas reported in earlier studies, the need for extending similar standardized studies for the *C. nutans* in other geographical regions is indicated.

## Figures and Tables

**Table 1:** Phytochemical contents of C. nutans based on different origin and extraction method.

Compound	Origin	Plant part	Extract solution	Present/Absent	Reference
Phenolic compounds	Malaysia	Leaf	Methanol (100%)	Present	[17]
Flavonoids	Malaysia	Leaf	Methanol (100%)	Present	[8,17,18]
	Indonesia	Leaf	Methanol (70%)	Present	[8,19]
		Leaf	Water	Present	[20]
Alkaloids	Malaysia	Leaf	Methanol (100%)	Absent	[8,17,18]
	Indonesia	Leaf	Methanol (70%)	Absent	[8,19]
		Leaf	Aqueous methanol	Absent	[8]
		Leaf	Water	Present	[20]
Saponins	Malaysia	Leaf	Methanol (100%)	Present	[8,17,18]
	Indonesia	Leaf	Methanol (70%)	Absent	[8,19]
		Leaf	Chloroform	Absent	[8]
		Leaf	Aqueous methanol	Absent	[8]
		Leaf	Water	Absent	[20]
Triterpenoids	Malaysia	Leaf	Methanol (100%)	Present	[8,18]
	Indonesia	Leaf	Water	Present	[20]
Diterpenes	Malaysia	Leaf	Methanol (100%)	Present	[17]
	Indonesia	Leaf	Water	Present	[20]
Steroids	Malaysia	Leaf	Methanol (70%)	Present	[8,19]
	Thailand	Leaf	Methanol (100%)	Present	[8,18]
		Stem	Light petroleum	Present	[21]
Phytosterols	Malaysia	Leaf	Methanol (100%)	Present	[8,17]
		Leaf	Methanol (70%)	Present	[8,19]
Tannins	Malaysia	Leaf	Methanol (100%)	Absent	[17,18]
	Indonesia	Leaf	Methanol (70%)	Present	[19]
		Leaf	Water	Present	[20]
Carbohydrates	Malaysia	Leaf	Methanol (70%)	Present	[19]
Protein/amino acids	Malaysia	Leaf	Methanol (70%)	Present	[19]

**Table 2:** Different metabolites identified in C. nutans.

Plant part	Compound	Class	Reference
Leaf buds	Gallic acid	Phenolic acid	[22]
	Caffeic acid	Phenolic acid	[22]
	Kaempferol	Flavonoids	[22]
	Catechin	Flavonoid	[22]
	Luteolin	Flavonoid	[22]
	Quercetin	Flavonoid	[22]
Leaf	Gallic acid	Steroid	[22]
	Caffeic acid	Phenolic acid	[22]
	Kaempferol	Flavonoid	[22]
	Catechin	Flavonoid	[22]
	Luteolin	Flavonoid	[22]
	Quercetin	Flavonoid	[22]
	Isovitexin	*C*-glycosidic flavone	[15]
	Schaftoside	*C*-glycosidic flavone	[15]
	Isoorientin	*C*-glycosidic flavone	[15]
	Orientin	*C*-glycosidic flavone	[15]
	Vitexin	*C*-glycosidic flavone	[15]
	Clinacoside B	Sulphur-containing glucoside	[23]
	Clinacoside C	Sulphur-containing glucoside	[23]
	Cycloclinacoside A1	Sulphur-containing glucoside	[23]
	Cycloclinacoside A2	Sulphur-containing glucoside	[23]
	Clinamide A	Sulphur-containing compound	[24]
	Clinamide B	Sulphur-containing compound	[24]
	Clinamide C	Sulphur-containing compound	[24]
	2-cis-entadamide A 1-O-β-D-glucosides of phytosphingosines	Sulphur-containing compound	[24]
		Cerebroside	[25]
	(2S)-1-O-linolenoyl-3-O-β-D-galactopyranosylglycerol	Glycosylglyceride	[25]
	13^2^-hydroxy-(13^2^-R)chlorophyll	Chlorophyll derivative	[26]
	*b* 13^2^-hydroxy-(13^2^-S)phaeophytin a	Chlorophyll derivative	[26]
	13^2^-hydroxy-(13^2^-S)phaeophytin	Chlorophyll derivative	[26]
	b 13^2^-hydroxy-(13^2^-R)chlorophyll	Chlorophyll derivative	[26]
	*b* 13^2^-hydroxy-(13^2^-S)chlorophyll *b*	Chlorophyll derivative	[26]
	Purpurin 18 phytyl ester	Chlorophyll derivative	[26]
	Phaeophorbide a 1,2-benzenedicarboxylic acid, mono(2-ethylhexyl) ester	Chlorophyll derivative	[26]
		Benzenoid	[26]
Stem	Stigmasterol β-sitosterol	Steroid	[27]
	Lupeol	Steroid	[28]
	Vitexin	Triterpenoid	[28]
	Clinacoside A	C-glycosyl flavones	[29]
		Sulphur-containing glucoside	[23]
Root	β-sitosterol	Steroid	[30]
